# ADVANCING HOSPICE CARE: A SCOPING REVIEW OF DECISION-SUPPORT TOOLS IN END-OF-LIFE

**DOI:** 10.1093/geroni/igae098.2381

**Published:** 2024-12-31

**Authors:** Susanny Beltran, Shanelle Harvey

**Affiliations:** University of Central Florida, Orlando, Florida, United States; University of Central Florida, Orlando, Florida, United States

## Abstract

Hospice, known for improving end-of-life (EOL) care outcomes and reducing hospitalizations and medical expenses, faces challenges with eligibility and referral processes. This often leads to late referrals and short lengths of stays which undermine patients’ ability to fully benefit from the service. In the era of electronic medical records, decision-support tools (DST) offer a promising solution for enhancing decision-making in healthcare. This scoping review, guided by Arksey and O’Malley’s framework and the Joanna Briggs Institute guidelines, maps the use of DSTs in EOL care, and reports their application (i.e., diagnoses, interventions, facilitators and barriers, and efficacy). Our search in EBSCOhost databases like MEDLINE, PsycInfo, and CINAHL, from June to September 2022 (updated July 6, 2023), used terms related to EOL care and DSTs. From 624 identified studies, 76 were considered, and 30 met the inclusion criteria upon full review. DSTs for EOL care have evolved into two primary categories: Prognostic Tools (PT), focusing on conditions like heart failure, dementia, and COPD, using clinical indicators; and Clinical Decision Support tools (CDS), aiding assessment, and referral in palliative care with techniques like structured interviews and natural language processing. The 30 studies revealed broad application across various illnesses. Barriers commonly reported included limited generalizability of the research, and late referrals. Facilitators include collaborative initiatives, user-friendly interfaces, integration into clinical workflows, and training for healthcare providers. This diverse array of tools and their varied applications highlight the complexities and potential in EOL care, emphasizing the need for improving their integration to enhance care outcomes.

